# A nationwide questionnaire survey of physicians regarding the impact of the COVID-19 pandemic on patients and treatment system of psychosomatic medicine

**DOI:** 10.1186/s13030-023-00279-0

**Published:** 2023-06-08

**Authors:** Yukari Yamanaka, Kazuhiro Yoshiuchi, Chiharu Kubo, Shin Fukudo, Japanese Society of Psychosomatic Medicine, Ichiro Kusumi, Ken Sato, Shoichi Ebana, Keisuke Kawai, Takeaki Takeuchi, Mutsuhiro Nakao, Masahiro Hashizume, Shuichiro Maruoka, Hiroshi Kaneko, Yasuhiro Kawasaki, Mikihiko Fukunaga, Atsuko Koyama, Makoto Hashizume, Hiroki Okada, Toshihide Harada, Nobuyuki Sudo, Akihiro Asakawa, Sunao Matsubayashi

**Affiliations:** 1 Department of Stress Sciences and Psychosomatic Medicine, Graduate School of Medicine, The University of Tokyo, 7-3-1 Hongo, Bunkyo-ku, Tokyo, Japan; 2 Nakamura Gakuen University, 5-7-1 Befu, Jonan-ku, Fukuoka City, Fukuoka Japan; 3 Department of Behavioral Medicine, Tohoku University Graduate School of Medicine, 2-1 Seiryo, Aoba, Sendai, Japan

**Keywords:** COVID-19, Questionnaire survey, Psychosomatic medicine, Japan

## Abstract

**Background:**

The coronavirus disease 2019 (COVID-19) pandemic has affected the treatment system of medical institutions across the world. Studies of the populations and patients have reported mental health problems caused by the pandemic. However, there are few large-scale studies that have examined the effects of the COVID-19 on diseases from the perspective of psychosomatic medicine. The purpose of this study was to examine changes made to the psychosomatic treatment system of Japan during the COVID-19 pandemic and the impact of the pandemic on patients with diseases treated in psychosomatic medicine.

**Methods:**

We conducted a nationwide questionnaire survey of members of the Japanese Society of Psychosomatic Medicine and the Japanese Society of Psychosomatic Internal Medicine from December 24, 2021 to January 31, 2022.

**Results:**

Of the 325 respondents, 23% reported restrictions in initial outpatient admissions, 66% implemented telemedicine, 46% reported a decrease in outpatient admissions, and 31% working in facilities with inpatient units reported decreased inpatient admissions. To reduce in-person visits, 56% of the respondents decreased the frequency of patient visits and 66% introduced telemedicine. Seventy-eight percent of the respondents reported that the COVID-19 pandemic affected the onset or exacerbation of diseases treated in psychosomatic medicine, including psychosomatic disorders, anxiety disorders, mood disorders, adjustment disorders, and eating disorders.

**Conclusions:**

This study revealed that the COVID-19 pandemic might have affected the practice of psychosomatic treatment in Japan and that various alternative measures were taken to prevent infection. In addition, although the items in this study were not compared to pre-pandemic data, the COVID-19 pandemic, it could have significant psychosocial effects on Japanese patients requiring psychosomatic care. Furthermore, respondents believed that numerous psychosocial factors were behind the impact of the COVID-19 pandemic on patients with diseases treated in psychosomatic medicine.

**Supplementary Information:**

The online version contains supplementary material available at 10.1186/s13030-023-00279-0.

## Background

The coronavirus disease 2019 (COVID-19) pandemic affected medical institutions worldwide. In Japan, medical institutions and patients were forced to adapt to new conditions. Further, many medical institutions declined or deferred treatment, and patients themselves refrained from seeing their doctors because of pandemic-related concerns. The pandemic also led to changes that included infection prevention measures and the introduction of telemedicine to routine medical care.

A study of Japanese Society of Nephrology‑certified educational facilities reported that more than half of the facilities experienced a decrease in the number of inpatients and outpatients and that approximately 70% of the facilities introduced telemedicine [1]. However, there are no reports of a nationwide survey of Japanese facilities working in the field of psychosomatic medicine.

The impact of the COVID-19 pandemic on patients with psychosomatic disorders has been noted in several studies. A study of psychiatrists and psychologists for children and adolescents reported an increase in patients with depression, anxiety disorders, and psychosomatic disorders [2]. A population study found an association between the COVID-19 pandemic and psychosomatic symptoms [3].

The present study aimed to examine what changes have occurred in Japanese psychosomatic treatment in terms of outpatient and inpatient treatment system during the COVID-19 pandemic, what effects have occurred on disease onset and course of patients, and what psychosocial factors were related to these effects.

## Methods

A nationwide questionnaire survey was conducted among physician members of the Japanese Society of Psychosomatic Medicine (JSPM) and the Japanese Society of Psychosomatic Internal Medicine (JSPIM). These societies were selected because they represented Japanese societies involved with treating patients requiring psychosomatic care.

The authors of this study organized important points that could be used to evaluate the influence of the COVID-19 pandemic on usual treatment, disease onset and progression, and the number of patients with diseases treated with psychosomatic medical care, then developed an original questionnaire, referring to a study conducted in Japan in 2020 that used similar methods [1]. The English translation of the questionnaire is shown in a supplementary table. The questionnaire consisted of seven parts: (i) confirmation of consent and characteristics of the respondent, (ii) characteristics of the facility and department affiliation, (iii) implementation of infection prevention measures, (iv) influence of the COVID-19 pandemic on routine outpatient treatment, (v) influence of the COVID-19 pandemic on routine inpatient treatment, (vi) nosocomial infection in the affiliated department, and (vii) influence of the COVID-19 pandemic on disease onset and progression from the aspect of psychosomatic medical care. The questions about changes in usual treatment; (iii), (iv), and (v); were basically regarding the time at which the survey was given. The questions about nosocomial infection and the impact of the COVID-19 pandemic on patients; (vi) and (vii); were to be answered based on the respondents’ overall experiences from April 2020, when the first wave of infection spread.

The survey was conducted online using Google Forms. Invitation emails were sent to all members of JSPM and JSPIM. At the time of the survey, 1,827 medical doctors belonged to JSPM and 985 to JSPIM: 585 doctors belonged to both societies. The response period was from December 24, 2021 to January 31, 2022. During this period, the sixth wave of infection spread in Japan, and priority measures to prevent the spread of the disease were issued.

The study protocol was approved by the institutional review boards of JSPM and JSPIM, with consent implied by responding to the questionnaire.

We summarized the descriptive statistics from the responses.

## Results

### Respondent affiliations

The online questionnaire was completed by 325 physicians (response rate 14.6%), 233 (72%) of whom were psychosomatic physicians. Among them, 147 (45%) were internal medicine physicians other than psychosomatic physicians, 89 (27%) were psychiatrists, 15 (4.6%) were obstetricians and gynecologists, and 15 (4.6%) were pediatricians. The remaining 28 (8.6%) respondents represented palliative care, dermatology, surgery, urology, general medicine, otolaryngology, psycho-oncology, and rehabilitation. Some participants belonged to multiple departments. Among the respondents, 77 (24%) belonged to medical institutions officially designated for managing infectious diseases. The affiliations were as follows: clinics (40%), university hospitals (24%), general hospitals (20%), and hospitals other than university or general hospitals (16%).

### Implementation of infection prevention measures for COVID-19

According to the responses regarding outpatient settings, 221 (68%) left the door of the examination room partially or completely open, 109 (34%) placed partitions between physicians and patients, and 87 (27%) wore goggles or face shields at all times. The vast majority (n = 312; 96%) reported they screened for diseases during clinic visits. The screening mostly comprised temperature measurement and symptom interview.

At the time of questionnaire response, 88% of the respondents working in facilities with inpatient units reported that they were implementing some form of COVID-19 screening test for emergency admissions (polymerase chain reaction [PCR] by 32%, antigen tests by 12% chest computed tomography [CT] by 1.2%, or some combination of these tests by 43%) and 84% did so for scheduled admissions (PCR, antigen tests, chest CT, or some combination of these; 42%, 11%, 0.6%, and 31%, respectively).

### The influence of the COVID-19 pandemic on routine outpatient treatment

**Fig. 1:**
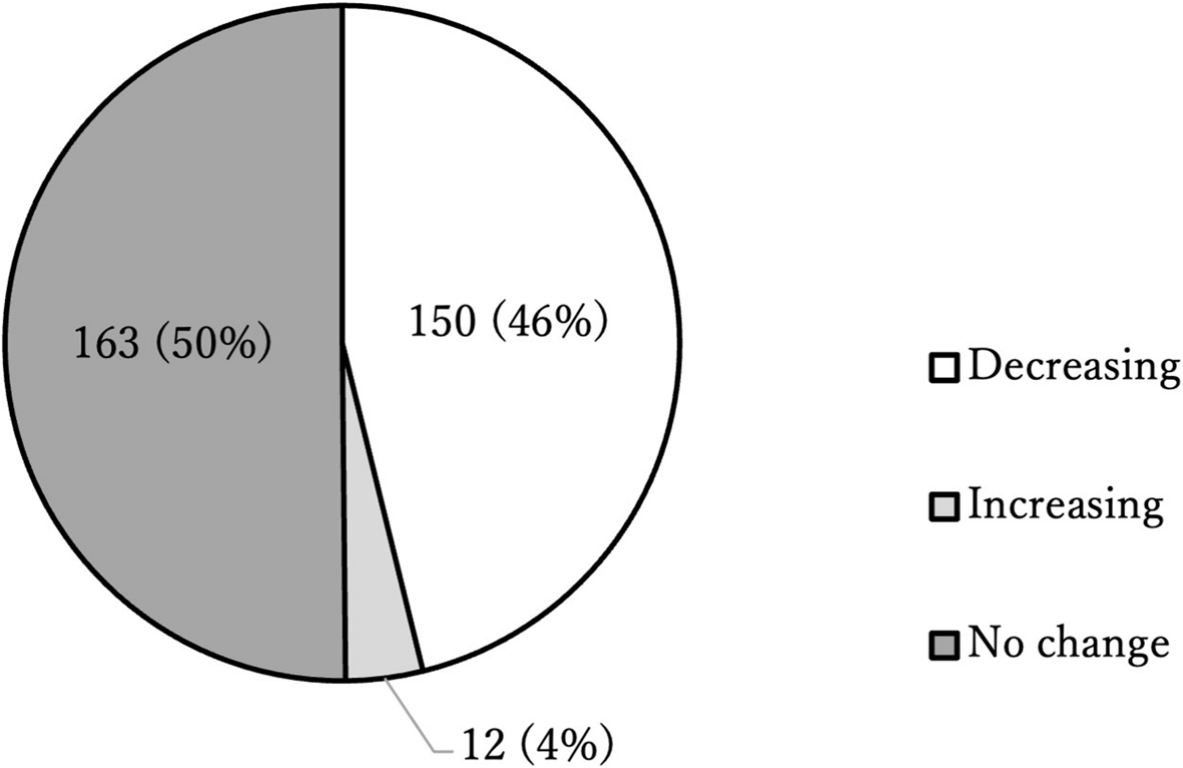
Responses about changes in the number of outpatients during the COVID-19 pandemic

The results of the responses about changes in the number of outpatients were divided into “decrease” and “no apparent change,” at 46% and 50%, respectively. A small number of respondents (3.7%) indicated that the number of outpatients increased during the pandemic (Fig. 1).

**Fig. 2:**
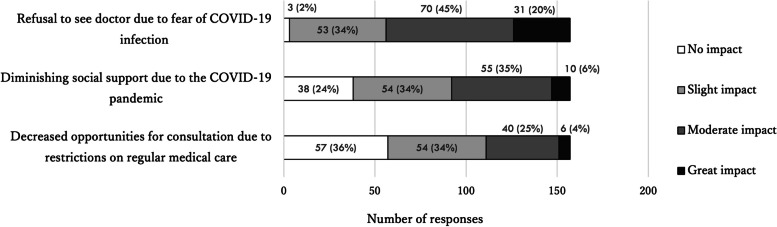
Responses about barriers to medical care during the COVID-19 pandemic

About half of the respondents (n = 157; 48%) answered that the COVID-19 pandemic had prevented some patients from accessing proper medical care. The most common reason reported was fear of contracting COVID-19, followed by decreased social support and restrictions on medical visits (Fig. 2).

Seventy-six respondents (23%) reported closure or reduction of outpatient services for first time visits during the COVID-19 pandemic. To decrease and minimize patient contact, about half of the respondents (56%) increased the interval between outpatient visits and 66% introduced telemedicine.

**Fig. 3:**
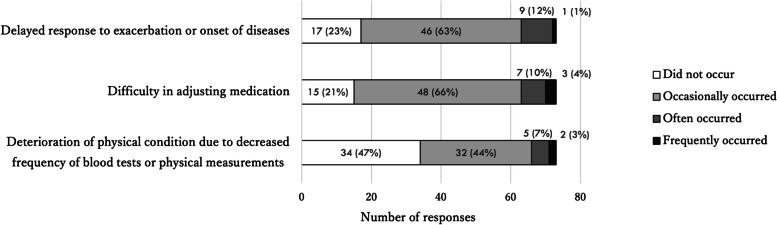
Responses about problems related to the reduced frequency of outpatient visits

**Fig. 4:**
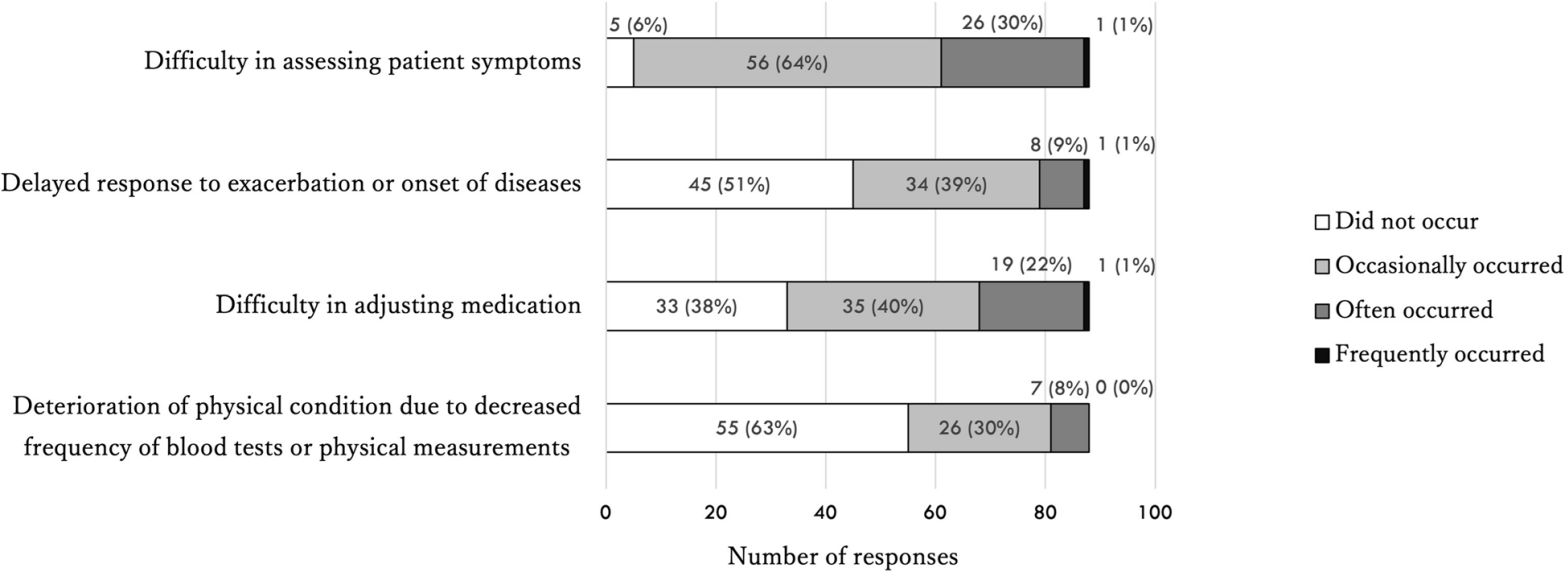
Responses about problems related to telemedicine services

Problems related to reduced frequency of patient visits and online/telephone consultations were reported by 72 (40%) and 88 (41%) of the respondents, respectively. The most frequently reported problems associated with less-frequent visits were delays in responding to exacerbation or onset of disease and difficulty in adjusting medication (Fig. 3). Almost all respondents who reported problems related to telemedicine felt that the remote modality made it difficult to accurately assess patient symptoms (Fig. 4).

### The influence of the COVID-19 pandemic on routine inpatient treatment

**Fig. 5:**
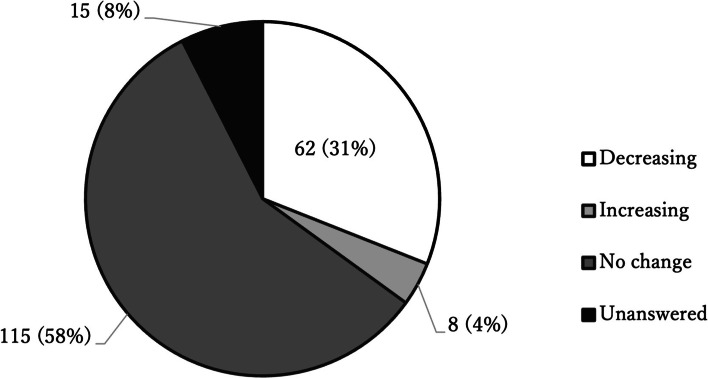
Responses about changes in the number of inpatients during the COVID-19 pandemic

Of all respondents, 200 who worked in facilities with inpatient units completed the questions in this section: 62 (31%) reported reduced inpatient visits, but a few (4.0%) reported an increase (Fig. 5); and 88 (44%) reported that their facilities limited inpatient care because of reductions in available beds brought about by the COVID-19 pandemic.

### Nosocomial COVID-19 infections in medical facilities

Twenty-five respondents (7.7%) reported nosocomial COVID-19 infection in their department. The number of persons who acquired nosocomial infections ranged from 1–100.

### The influence of the COVID-19 pandemic on disease onset and progression

**Fig. 6:**
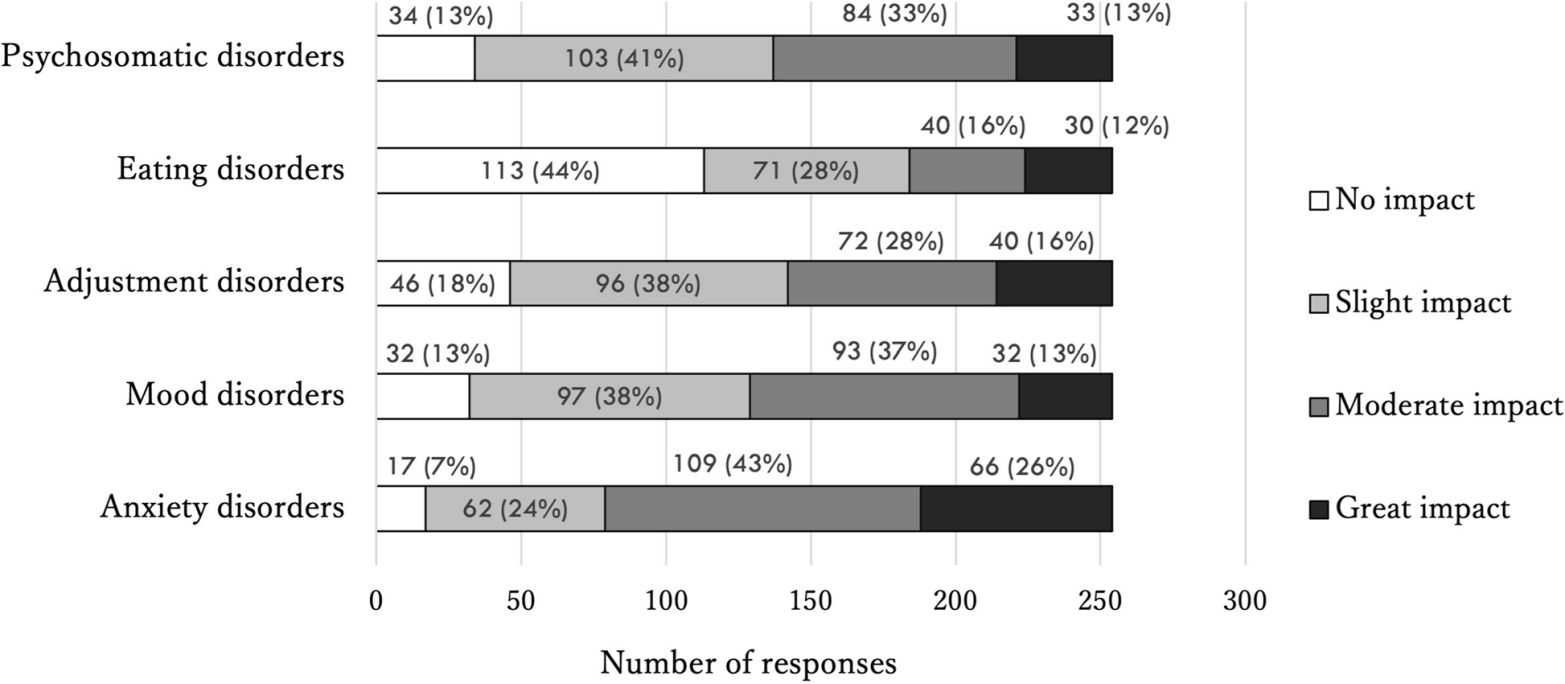
Responses about the impact of the COVID-19 pandemic on diseases treated in psychosomatic medicine

Two hundred and fifty-four respondents (78%) reported that the COVID-19 pandemic affected the onset and exacerbation of the diseases they treated. More than 90% of them reported that the COVID-19 pandemic affected patients with anxiety disorders and more than 80% reported adverse effects on patients with mood, psychosomatic, and adjustment disorders (Fig. 6).

In addition to the five diseases mentioned in the questionnaire, including the four previously mentioned diseases and eating disorders, multiple respondents felt that patients with schizophrenia, developmental disorder, somatic symptom disorder, dementia, lifestyle-related diseases, and skin diseases such as atopic dermatitis were affected by the COVID-19 pandemic.

**Fig. 7:**
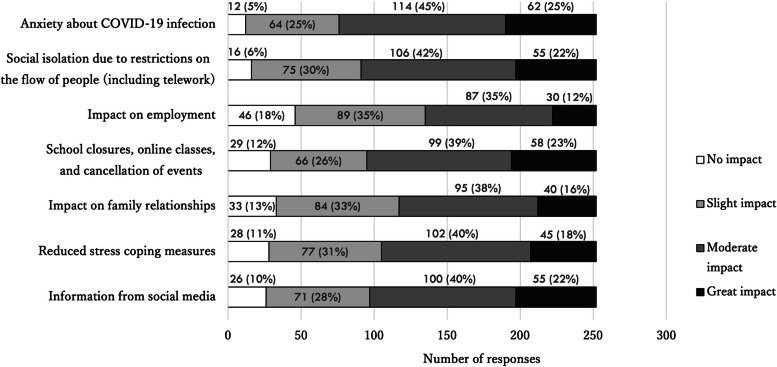
Responses about how the COVID-19 pandemic affected diseases treated in psychosomatic medicine

Figure 7 provides the responses as to why the COVID-19 pandemic affected patients with diseases treated in psychosomatic medicine. Respondents were asked about various factors, including individual issues such as anxiety about COVID-19 infection and diminished ways of coping with stress, and social issues such as social isolation due to restriction of the movement of people and changes in the schooling environment. For each of the factors presented in the questionnaire, more than 80% of the respondents answered that it had adversely affected their patients. Other responses included deteriorating economic conditions, lack of exercise due to self-restraint, decreased interaction with others, and avoiding medical examinations.

The respondents reported increases in the number of patients treated for anxiety or obsessive-compulsive disorders (173; 53%), depressive disorders (153; 47%), adjustment disorders (147; 45%), psychosomatic disorders (119; 37%), eating disorders (57; 18%), and severe anorexia nervosa requiring hospitalization (45; 14%).

## Discussion

This study is the first nationwide questionnaire survey of JSPM and JSPIM physicians to examine how the COVID-19 pandemic affected infection prevention measures, in- and outpatient treatment conditions, and patients with diseases treated with psychosomatic treatment.

Most respondents reported that their facilities attempted to prevent nosocomial infections via temperature checks, symptom surveys, and COVID-19 screening tests in inpatient settings. Some respondents also left their examination room doors open, wore goggles, and placed partitions between the patient and doctor.

About half of the respondents reported a reduced number of outpatients during the pandemic and a quarter of the respondents reported restrictions on initial outpatient visits. About 30% of the respondents from medical institutions with inpatient facilities reported a reduced number of inpatients. About half of the respondents extended the outpatient interval. Consequently, respondents reported difficulties in adjusting medications and delays in responding to symptom changes. Approximately 70% of the respondents provided online or telephone consultations. The most frequently reported problem associated with telemedicine was difficulty in assessing patient symptoms.

Most respondents indicated that the COVID-19 pandemic might have affected patients with diseases that required psychosomatic treatment. Approximately 40% reported an increased number of patients with psychosomatic disorders, adjustment disorders, depressive disorders, anxiety disorders, and obsessive-compulsive disorders. Some previous studies support this result and others do not. A longitudinal study conducted in a nationally representative sample in the US reported that anxiety symptoms increased markedly at the beginning of the COVID-19 pandemic [4]. Conversely, a meta-analysis of studies that compared the mental status of the populations pre- and post- the COVID-19 pandemic showed no change in anxiety and minimal worsening of depression symptoms, though female cohorts appear to have experienced worsening of anxiety and depression symptoms [5]. Prior psychiatric diagnoses, such as depression and anxiety, may predict the development of adverse psychological symptoms associated with lockdown [6, 7]. In addition, eating disorders (ED) seem to have become more prevalent since the beginning of the COVID-19 pandemic in Japan [8]. A change in the clinical features of Japanese ED patients after the first state of emergency (April 7, 2020) was that the age of first consultation was lower than that before the COVID-19 pandemic [9]. An Asian survey of physicians reported that irritable bowel syndrome and functional dyspepsia were greatly aggravated by the COVID-19 pandemic [10]. Although it is difficult to say which patient groups were more susceptible to the pandemic because of the varied backgrounds of the respondents in this survey, through their experience with patient examinations, physicians with expertise in psychosomatic medicine believe that the mental states of their patients have been influenced by the pandemic. Furthermore, previous studies reported that social isolation, economic challenges, and individual behavioral changes, such as decreased stress-coping behaviors and frequent use of social media, could worsen mental health problems associated with COVID-19 [11, 12]. Our study suggests that similarly, in the field of psychosomatic medicine in Japan, not only changes in the overall social conditions in the country, but also changes in family relationships and individual behavior patterns may have affected patients.

There are several limitations of this study. First, the response rate was low. The total number of respondents, 325, was small, even considering that a certain number of physicians did not work in the clinical field. Second, we are unable to provide details about the survey regions because we did not ask the location of the medical institutions or the academic branches to which the respondents belonged. Third, this survey was not targeted toward patients, but rather toward physicians, asking about their opinions and experiences with patients. Therefore, the responses to the questionnaire may not accurately reflect the actual situation of the patients. The extent of the increase in each disease and its background factors during the COVID-19 pandemic would be more accurately assessed by database and medical records or by conducting a questionnaire survey of patients. Lastly, this study is not a pre- and post- pandemic comparative study, therefore, we cannot discuss causality between the pandemic and the situation of the patients, as reported by the respondents.

## Conclusions

The COVID-19 pandemic affected the practice of psychosomatic medicine in Japan. The challenges they met prompted physicians to adapt by implementing appropriate infection prevention measures and introducing telemedicine services. The psychosocial impact of the COVID-19 pandemic may have contributed to the worsening and onset of diseases treated in the psychosomatic area. Future studies that also target patients should be conducted to more accurately investigate the impact of the pandemic on patients. It will also be important to consider mental health care related to the pandemic that can be applied to clinical practice given the ongoing nature of the COVID-19 pandemic.

## Supplementary Information

**Additional file 1: Supplementary table.** English translation of the questionnaire originally developed for this study.

## Data Availability

We cannot share our data because we did not obtain the consent of the respondents to share their data.
