# Supplementary material for: A nationwide questionnaire survey of physicians regarding the impact of the COVID-19 pandemic on patients and treatment system of psychosomatic medicine
Source: Biopsychosoc Med. 2023 Jun 8;17:21. doi: 10.1186/s13030-023-00279-0 (PMC10248960; doi:10.1186/s13030-023-00279-0)
Supplement: Supplementary file 1 — Additional file 1: Supplementary table. English translation of the questionnaire originally developed for this study. [file 13030_2023_279_MOESM1_ESM.docx]

Survey of the COVID-19 pandemic impact on treatment systems and patients in the field of psychosomatic medicine

[English translation]

(i) Confirmation of consent and characteristics of research subject

Are you a physician who is a member of the Japanese Society of Psychosomatic Medicine or the Japanese Society of Psychosomatic Internal Medicine, and currently engaged in a medical practice?

- Yes
- No

Do you understand the outline of this study and that it is impossible to withdraw your consent after completion of your response, and do you agree to participate in this survey?

- I agree to participate in this study.
- I do not agree to participate in this study.

(ii) Characteristics of respondents’ affiliated facilities and departments

1. Which society are you a member of? Please select all that apply.

- The Japanese Society of Psychosomatic Medicine
- The Japanese Society of Psychosomatic Internal Medicine

1. What is your department? Please select all that apply.

- Psychosomatic medicine
- Internal medicine other than psychosomatic medicine
- Psychiatry
- Obstetrics and gynecology
- Pediatrics
- Dermatology
- Other departments（　　　　　　　　　　　　　　　　　　　　　　）

1. Is your medical institution officially designated for managing infectious diseases?

- Yes
- No

1. Which is your medical institution?

- University hospital
- General hospital
- Hospital other than university or general hospital
- Clinic

1. What type of practice are you engaged in? Please select all that apply.

- Outpatient treatment
- Inpatient treatment
- Consultation Liaison

1. How many physicians are in your department?

　Full-time doctor（　　　　　）persons

　Part-time doctor（　　　　　）persons

1. Approximately how many patients per week does your department provide outpatient care?

（　　　　　）persons

1. Approximately how many patients are admitted to your department per day?

（　　　　　）persons

(iii) Implementation of infection prevention measures for COVID-19

1. Do all medical staffs always wear goggles or face shields during general practice?

- Yes
- No

1. Do you open the door of the examination room partially or completely?

- Yes
- No

1. Do you implement partitions between you and patients?

- Yes
- No

1. Do you screen for infections by taking body temperature and interviewing patients about their symptoms when they arrive at the hospital?

- Yes
- No

Please select all applicable infection prevention measures at the time of admission that are in place at the time of your response.

1. Scheduled admission cases

- PCR test
- Antigen test
- Chest CT
- None of the above

1. Emergency admission cases

- PCR test
- Antigen test
- Chest CT
- None of the above

Please select all applicable infection prevention measures at the time of admission that have ever been taken.

1. Scheduled admission cases

- PCR test
- Antigen test
- Chest CT
- None of the above

1. Emergency admission cases

- PCR test
- Antigen test
- Chest CT
- None of the above

(iv) The influence of the COVID-19 pandemic on outpatient treatment

1. Has the number of outpatients in your department changed?

- Yes
- No

1. Please answer if you answered "Yes" to the previous question. If pre-pandemic COVID-19 was 100%, what is the approximate percentage?

（　　　　　）%

1. Have you experienced closure or reduction of outpatient services for first time visits during the COVID-19 pandemic?

- Outpatient services for first time visits have been closed after the COVID-19 pandemic
- Outpatient services for first time visits have been reduced after the COVID-19 pandemic
- Outpatient services for first time visits were closed temporarily.
- Outpatient services for first time visits were reduced temporarily.
- No closure or reduction

1. Do you keep intervals between outpatient visits longer than usual?

- Yes
- No

1. Have you introduced online or telephone medical care?

- Yes
- No

1. Have you experienced any problems related to reduced frequency of patient visits?

- Yes
- No

1. Please answer if you answered "Yes" to the previous question. Please select all that apply and tell us the extent to which each of the following has had an impact on your practice.

- Delayed response to exacerbation or onset of diseases

　□Occasionally occurred　□Often occurred　□Frequently occurred

- Difficulty in adjusting medication

　□Occasionally occurred　□Often occurred　□Frequently occurred

- Deterioration of physical condition due to decreased frequency of blood tests or physical measurements

　□Occasionally occurred　□Often occurred　□Frequently occurred

- Other problems（　　　　　　　　　　　　　　　　　　　　　　　　　　 ）

　□Occasionally occurred　□Often occurred　□Frequently occurred

1. Have you experienced any problems related to online/telephone consultation?

- Yes
- No

1. Please answer if you answered "Yes" to the previous question. Please select all that apply and tell us the extent to which each of the following has had an impact on your practice.

- Difficulty in assessing patient symptoms

　□Occasionally occurred　□Often occurred　□Frequently occurred

- Delayed response to exacerbation or onset of diseases

　□Occasionally occurred　□Often occurred　□Frequently occurred

- Difficulty in adjusting medication

　□Occasionally occurred　□Often occurred　□Frequently occurred

- Deterioration of physical condition due to decreased frequency of blood tests or physical measurements

　□Occasionally occurred　□Often occurred　□Frequently occurred

- Other problems（　　　　　　　　　　　　　　　　　　　　　　　　　　　）

　□Occasionally occurred　□Often occurred　□Frequently occurred

(v) The influence of the COVID-19 pandemic on routine inpatient treatment

1. Does your facility have an inpatient unit?

- Yes
- No

1. How many inpatient beds does your facility have in total?

（　　　　　）beds

1. If your facility has an inpatient unit for COVID-19 patients, how many beds does it have?

（　　　　　）beds

1. How many beds does your department have?

（　　　　　）beds

1. Has the number of inpatients in your department changed after the COVID-19 pandemic?

- Yes
- No

1. Please answer if you answered "Yes" to the previous question. If pre-pandemic COVID-19 was 100%, what is the approximate percentage?

（　　　　　）%

1. Have there been any cases in which inpatient treatment in your department was postponed due to the COVID-19 pandemic?

- Yes
- No

(vi) Nosocomial infection in your department

1. Have there been any cases of COVID 19 nosocomial infections among staffs or patients in your department?

- Yes
- No

1. Please answer if you answered "Yes" to the previous question. How many people in total were infected due to nosocomial infections?

（　　　　　）persons

1. How many of them were staff members?

（　　　　　）persons

(vii) Influence of the COVID-19 pandemic on disease onset and progression in the area of psychosomatic medicine

1. Have you experienced any cases in which the COVID-19 pandemic may have influenced the onset or worsening of disease?

- Yes
- No

1. Please answer if you answered "Yes" to the previous question. What diseases do you think the COVID-19 pandemic affected? Please select all that apply and tell us the extent of the pandemic’s impact on each disease.

- Psychosomatic disorders

　□Slight impact　□Moderate impact　□Great impact

- Eating disorders

　□Slight impact　□Moderate impact　□Great impact

- Adjustment disorders

　□Slight impact　□Moderate impact　□Great impact

- Mood disorders

　□Slight impact　□Moderate impact　□Great impact

- Anxiety disorders

　□Slight impact　□Moderate impact　□Great impact

- Other disorders（　　　　　　　　　　　　　　　　　　　　　）

　□Slight impact　□Moderate impact　□Great impact

1. What factors do you think contributed to the impact of the COVID-19 pandemic on the patients? Please select all that apply and tell us the extent of the pandemic’s impact of each.

- Anxiety about COVID-19 infection

　□Slight impact　□Moderate impact　□Great impact

- Social isolation due to restrictions on the flow of people (including telework)

　□Slight impact　□Moderate impact　□Great impact

- Impact on employment

　□Slight impact　□Moderate impact　□Great impact

- School closures, online classes, and cancellation of events

　□Slight impact　□Moderate impact　□Great impact

- Impact on family relationships

　□Slight impact　□Moderate impact　□Great impact

- Reduced stress coping measures

　□Slight impact　□Moderate impact　□Great impact

- Information from social media

　□Slight impact　□Moderate impact　□Great impact

- Other factors (　　　　　　　　　　　　　　　　　　　　　　)

　□Slight impact　□Moderate impact　□Great impact

1. Were there any diseases for which the number of patients increased after the COVID-19 pandemic? Please select all that apply and tell us the extent of the increase.

- Psychosomatic disorders　（　　　　　）%
- Eating disorders　（　　　　　）%
- Severe anorexia nervosa requiring hospitalization 　（　　　　　）%
- Adjustment disorders　（　　　　　）%
- Mood disorders　（　　　　　）%
- Anxiety and obsessive-compulsive disorders　（　　　　　）%
- Other disorders（　　　　　　　　　　　　　　　　　　　　　）　（　　　　　）%

1. Have you experienced any cases in which the COVID-19 pandemic had prevented from accessing proper medical care?

- Yes
- No

1. Please answer if you answered "Yes" to the previous question. Please select all that apply and tell us the extent of the pandemic’s impact on each reason.

- Refusal to see doctor due to fear of COVID-19 infection

　□Slight impact　□Moderate impact　□Great impact

- Diminishing social support due to the COVID-19 pandemic

　□Slight impact　□Moderate impact　□Great impact

- Decreased opportunities for consultation due to restrictions on regular medical care

　□Slight impact　□Moderate impact　□Great impact

- Other reasons (　　　　　　　　　　　　　　　　　　　　　　　)

　□Slight impact　□Moderate impact　□Great impact

1. Please let us know if you have noticed any impact of the COVID-19 pandemic on your department's treatment systems or on your patients.
